# The characteristics and risk factors for common psychiatric disorders in patients with cancer seeking help for mental health

**DOI:** 10.1186/s12888-019-2251-z

**Published:** 2019-09-03

**Authors:** Dilek Anuk, Mine Özkan, Ahmet Kizir, Sedat Özkan

**Affiliations:** 1 Department of Consultation Liaison Psychiatry, Department of Psychiatry, Istanbul Faculty of Medicine, Istanbul University, Capa, 34390 Istanbul, Turkey; 2 Department of Radiation Oncology, Institute of Oncology, Istanbul University, 34390 Istanbul, Capa Turkey

**Keywords:** Help-seeking, Mood disorders, Adjustment disorders, Anxiety disorders, Psycho-oncology, Outpatients, Recurrence

## Abstract

**Background:**

Although the adverse effects of cancer diagnoses and treatments on mental health are known, about less than 10% of patients are estimated to be referred to seek help. The primary purpose of this study was to obtain the baseline information on patients with cancer seeking help for mental health who presented for the first time to the psycho-oncology outpatient clinic, and to identify risk factors that may provide clues healthcare practitioners in recognizing those needing psychological help in oncology practice.

**Methods:**

We reviewed the charts of 566 patients with cancer who were referred to the psycho-oncology outpatient clinic over a two-year period. The study includes the socio-demographic data, illness characteristics, psychiatric characteristics, psychiatric diagnoses, and treatment recommendations for these patients.

**Results:**

The incidence of diagnoses of psychiatric disorders was 97.5%. The distributions of psychiatric diagnoses were as follows: any kind of adjustment disorders, mood disorders, anxiety disorders, organic brain syndrome, personality disorders, delusional disorder, and insomnia. Recurrence of cancer, other chronic medical illnesses, a history of psychiatric disorders, poor social support, and low income comprised the common significant risk factors for adjustment disorders, mood disorders, and anxiety disorders. These risk factors were also seen to be significant in the regression analysis in terms of sex.

**Conclusion:**

This study identifies the distribution of psychiatric disorders, the risk factors for specific psychiatric disorders, and draws attention to the fact that there are serious delays in patients seeking psychiatric help and in the referrals of oncologists for psychological assessment. Identifying risk factors and raising oncologists’ awareness toward risk factors could help more patients gain access to mental health care much earlier.

## Background

Cancer is the leading cause of deaths in developed countries and second after heart disease in developing countries [1]. Despite improvements in treatment, cancer is still believed to have a high mortality rate, and is considered to have an inherent potential for death, suffering and pain, causing considerable psychological distress, even in patients with a high recovery rate [2]. Its diagnosis poses a crisis requiring the patient to comply with a number of tremendous challenges. While trying to make serious treatment decisions, patients try to cope with intense emotional stress [3]. The emotional distress includes the diagnosis of a life-threatening illness, aggressive medical treatment, changes in lifestyle or direct effects of the tumor itself, lack of family support system, personality traits, familial conflicts, and economic problems [4–6].

The incidence of psychological disorders in patients with cancer is very high (30–60%) [7–9], with approximately 29–43% fulfilling the diagnostic criteria for psychiatric disorders [10, 11]. The most commonly encountered mental problems encompass depressive symptoms associated with mixed anxiety and adjustment disorder or depressive mood or major depression [7, 11]. Razavi et al. identified 47% of psychiatric diagnoses of those attending both outpatient and inpatient departments [12]. Kissane et al. showed that psychiatric diseases were diagnosed in 73% (24% systemic family problems, 23% mood disorders, 16% adjustment disorders, and 10% organic mental disorders) of 271 patients with cancer referred to Consultation Liaison Psychiatry [13]. A total of 765 patients with cancer referred to a psycho-oncology unit in Japan had diagnoses of psychiatric diseases, 59 (6%) of whom were outpatient patients, including adjustment disorders (24%), delirium (16%), and major depressive disorder (12%) [10].

Left untreated, psychological distress would lead to long-term devastating consequences with regard to non-compliance with treatment [14], low survival rates [15], desire to accelerate death [16], and poor quality of life for both patients and their relatives [17, 18].

Although adverse effects of cancer diagnoses and treatments have long been recognized, it is estimated that less than 10% of patients are referred to seek psychological help [19]*.* The primary purpose of this study was to obtain baseline information on psychological help-seeking in patients with cancer who presented for the first time to the psycho-oncology outpatient clinic.

The answers to two crucial research questions were sought:

- What were the socio-demographic, cancer-related as well as psychiatric characteristics of patients with cancer seeking help who presented to the psycho-oncology outpatient clinic?
- What were both the overall and sex-based risk factors in the development of mood disorders, adjustment disorders, and anxiety disorders in those patients?

## Methods

### Design

This study was conducted at the Oncology Institute of Istanbul University, one of the leading hospitals in the field in Turkey where, on average, 5000 new patients present per year and 60,000 patients are followed up at 3, 6 months, and 12 months after treatment. Patients with cancer and their relatives receive psychiatric and psychological treatment services at the Istanbul University Consultation Liaison Psychiatry Department. A multidisciplinary team (2 psychiatrists, 1 psychologist) provides this service at the psycho-oncology outpatient clinic in the Oncology Institute building, with about 800 (inpatient and outpatient) patients annually. This is an exploratory study with a retrospective chart review design. We reviewed the charts of 566 patients with cancer who were referred to the psycho-oncology outpatient clinic from January 2015 to February 2017. The sociodemographic data, psychiatric characteristics, psychiatric diagnoses, and treatment recommendations in the patients’ charts were assessed. Data on the diseases and psychiatric diagnoses were established based on the Diagnostic and Statistical Manual of Mental Disorders, 4th Edition (DSM-IV-TR) [20]. The study was reviewed and approved by the institutional review boards at the Oncology Institute of Istanbul University in terms of ethics (No: 709731125–604.01.01). Due to the fact that the study was designed as a retrospective analysis and we only reviewed the patients’ charts without performing interviews or direct evaluations with the patients, it was not necessary to obtain written consent from the patients. All patients’ records were anonymized and de-identified prior to analyses.

### Data analyses

The statistical analysis was performed using the SPSS 21.0 statistical package program. The demographic and clinical data of the participants were analyzed using descriptive statistics. Pearson’s Chi-square test and Fisher’s exact test were used for the comparison of qualitative data. Logistic regression analysis was used to examine the risk factors for adjustment disorders, mood disorders, and anxiety disorders. A regression analysis model was developed including the sociodemographic characteristics (age, sex, education, income level, occupation), medical condition-related characteristics (duration of illness, presence of recurrence, presence of metastasis, presence of secondary cancer, and other chronic illnesses), and psychiatric variables (social support, history of psychiatric disorders or family history of psychiatric disorders) to identify significant risk factors for the three psychiatric groups, i.e. mood disorders, adjustment disorders, and anxiety disorders. Regression analysis was first performed for all patients and then for the sexes using the same model excluding sex. In the logistic regression analysis, variables were selected by the enter method and the risk ratios [odds ratio (OR)] were calculated by taking the first categories as references. The results were assessed at 95% confidence intervals, and *p* < 0.05 as the significance level. Only significant risk factors are included in the tables in the regression analysis.

## Results

### Sociodemographic and Cancer-related characteristics of help-seeking patients

The rate of women presenting to the psycho-oncology outpatient clinic (62%) was higher than for men (38%) (χ^2^: 32.678 / *p* < 0.001). The mean age of the women was 49.62 ± 13.4 years, which was statistically significantly lower than in the men (53.83 ± 16 years) (t: 3.370, *p* = 0.001). Of the patients, 76% were married and 90.5% were living in nuclear family conditions. The educational status was as follows: mostly secondary school graduates (55.5%) and literate / elementary school graduates (33.2%), and college graduates (11.3%); 37.5% were housewives, 31.1% were retired, 26.9% were working, and 4.6% were students. Three-quarters (76%) of the patients had middle-income status, 51.1% had poor social support, 82.7% lived in urban areas, and all benefited from social security services.

The oncologic diseases, which lasted between 1 month and 240 months (Mean ± SD: 22.3 ± 33.1 months), included breast cancer (31.1%), lung cancer (11.1%), gastrointestinal cancers (12.4%), head and neck cancer (16.6%), and gynecologic cancers (9.9%). Metastatic involvement was present in 22.6% of the patients, 20.1% had cancer recurrence, and 2.8% had secondary cancer. Of all patients, 20% also had chronic medical illnesses such as diabetes and hypertension, and their first-degree relatives (14.5%) had had any type of cancer diagnosis (Table 1).

**Table 1:** Sociodemographic and cancer-related characteristics of help-seeking patients

*Age* (years): Mean ± SD (Range)		
Overall Patients	51.22 ± 14.55 (16–87)	
Female (t: 3.370, *p* = 0.001)	49.62 ± 13.4 (16–85)	
Male	53.83 ± 16 (16–87)	
	n	%
*Sex*		
Female (χ^2^: 32.678/ p < 0.001)	351	62
Male	215	38
*Education level*		
Literate / elementery school	314	55.5
Secondary school graduates	188	33.2
College	64	11.8
*Marital status*		
Married	430	76
Single	59	10.4
Divorced/Widowed	77	13.6
*Employment*		
Housewife	212	37.5
Working fulltime	152	26.9
Retired	176	31.1
Other	26	4.6
*Income level*		
Low	120	21.1
Middle	430	76
Upper	16	2.8
*Type of family*		
Core	512	90.5
Extended	40	7.1
Alone	14	2.5
*Cancer site*		
Breast	176	31.1
Lung	63	11.1
Gastrointestinal cancers	70	12.4
Head and neck cancer	64	11,3
Brain tumors	30	5.3
Gynecologic cancers	56	9.9
Male genitourinary cancers	29	5.1
Leymphoma	36	6.4
Malignant melanoma /skin cancer	12	2.1
Bone and soft tissue cancer	17	3
Other	13	2.3
*Stage*		
Local disease	315	55.7
Locoregional recurrence	114	20.1
Metastatic	128	22.6
Unknown	9	1.6
*Oncological treatment*		
Surgery+chemotherapy+ radiotheraphy (+hormone therapy)	182	32.2
Surgery+chemotherapy	112	19.8
Chemotherapy+ radiotheraphy	85	15
Chemotherapy	76	13.4
Surgery+radiotheraphy	51	9
Radiotheraphy	19	3.4
Surgery+chemotherapy+hormone therapy	16	2.8
Surgery	12	2.1
Surgery +interferon	3	0.5
Treatment is not yet started	4	0.7
Other	9	1.6
*Any other medical illnesses*		
Yes	113	20
No	453	80
*Family history of cancer*		
Yes	82	14.5
No	484	85.5
*Secondary cancer*		
Yes	16	2.8
No	550	97.2
*Duration of cancer* (month): Mean ± SD (Range)	22.3 ± 33.1 (1–240)	

### Psychiatric characteristics and distribution of diagnoses

The majority (81.6%) of the patients were referred by attending oncologists, 9.5% were brought by family members, and 8.8% presented on their own to the outpatient department of psycho-oncology. Sleep problems, irritability, tendency to cry easily, sadness, and pain were among the leading symptoms at baseline. Women reported sleep problems, tendency to cry easily, irritability, pre-occupation with the illness, and sadness as the first five most frequent issues, and men reported sleep problems, irritability, pain (usually incompatible with their medical conditions), sadness, and tendency to cry easily as the most frequent problems (Table 2).

**Table 2:** Psychiatric characteristics of the patients

	Female		Male		Total	
	n	%	n	%	n	%
*Who is requesting the application*						
By the oncologists	293	83.5	169	78.6	462	81.6
By ownself	41	11.7	9	4.2	50	8.8
By family members	17	4.8	37	17.2	54	9.5
*Referral symptoms*						
Sleep problems	92	26.2	84	39.1	176	31.1
Tendency to cry easily	74	21.1	26	12.1	100	17.7
Irritability / intolerance	67	19.1	56	26	123	21.7
Despondency	48	13.7	27	12.6	75	13.3
Pre-occupation with illness	63	17.9	22	10.2	85	15
Trouble / restlessness	35	10	26	12.1	61	10.8
Pessimism	14	4	6	2.8	20	3.5
Olm / metastasis / fear of recurrence	14	4	4	1.9	18	3.2
Palpitations, fainting sensation	14	4	2	0.9	16	2.8
Marital problem	25	7.1	2	0.9	27	4.8
Pain	41	11.7	29	13.5	70	12.4
Inability to enter MR or the radiotherapy device	16	4.6	9	4.2	25	4.4
Concern	39	11.1	16	7.4	55	9.7
Unhappiness	12	3.4	7	3.3	19	3.4
Precision / obsession	4	1.1	1	0.5	5	0.9
Forgetfulness	7	2	6	2.8	13	2.3
Body image problems	14	4	5	2.3	19	3.4
Control of a previously started drug	6	1.7	4	1.9	10	1.8
Getting newly cancer diagnosis	3	0.9	3	1.4	6	1.1
Treatment rejection	3	0.9	5	2.3	8	1.4
Request for support	12	3.4	2	0.9	14	2.5
Other	6	1.7	6	2.8	12	2.1
No complaints	11	3.1	13	6	24	4.2
*Psychiatric Diagnosis*						
Anxiety disorders						
Anxiety disorder	19	5.4	8	3.7	27	4.8
Common anxiety disorder	9	2.6	4	1.9	13	2.3
Panic disorder	2	0.6	1	0.5	3	0.5
The claustrophobia	4	1.1	–	–	4	0.7
Obsessive-compulsive disorder	1	0.3	–	–	1	0.2
Somatoform Disorder	5	1.4	2	0.9	7	1.2
Mood disorders						
Depression	58	16.5	27	12.6	85	15
M depression + obsessive compulsive disorder	5	1.4	–	–	5	0.9
M depression + generalized anxiety disorder	3	0.8	4	1.9	7	1.2
Complicated grief reaction	2	0.6	–	–	2	0.4
Bipolar disorder	2	0.6	–	–	2	0.4
Adjustment Disorders						
Anxious adjustment disorder	112	31.9	94	43.7	206	36.4
Depressive adjustment disorder	94	26.8	49	22.8	143	25.2
Mixt adjustment disorder	16	4.6	3	1.4	19	3.4
Delirium	2	0.6	6	2.8	8	1.4
Insomnia	1	0.3	4	1.9	5	0.9
Personality disorder	3	0.9	5	2.3	8	1.4
Delusional disorder	4	1.1	3	1.4	7	1.2
No complaints	9	2.6	5	2.3	14	2.5
*History of psychiatric disorder*						
Yes	28	8	15	7	43	7.6
No	323	92	200	93	523	92.4
*History of psychiatric disorder in family*						
Yes	2	0.6	2	0.9	4	0.7
No	349	99.4	213	99.1	562	99.3
*Social Support*						
Sufficient social support	168	47.9	109	50.7	277	48,9
Poor social support	183	52.1	106	49.3	289	51.1
*Psychiatric Treatment Recommendation*						
Medication	207	59	152	70.7	359	63.4
Medication + psychotherapy	85	24.2	30	14	115	20.3
Psychotherapy	49	14	26	12.1	75	13.2
Follow-up without medication	10	2.8	5	2.3	15	2.7
Directing to the Alcohol Substance unit	–	–	2	0.9	2	0.4

The rate of psychiatric diagnoses was 97.5% and the distribution of psychiatric diagnoses was as follows: any kind of adjustment disorders (65%), mood disorders (18%), anxiety disorders (9.7%), organic brain syndrome (1.4%), personality disorders (1.4%), delusional disorder (1.2%), and insomnia 0.9%. The comorbid diagnosis was made in 2.1% (depression accompanied by anxiety disorder). Only 2.5% of the patients had no psychiatric diagnosis. Previous psychiatric treatment had been received by 7.6% of the patients, and 0.7% had a family history of mental illnesses. Just under half (48.9%) of patients had adequate social support, 51.1% poor social support (Table 2).

### Risk factors for mood disorders, adjustment disorders, and anxiety disorders

Significant risk factors for diagnosis of mood disorders included recurrence of cancer, other chronic medical illnesses, history of psychiatric disorder, secondary cancer presence, metastasis, poor social support, low income level, being single or divorced, and low educational level. This model explained 57% of the total variance, being useful to predict the evolution of mood disorders (Table 3).

**Table 3:** Risk factors for mood disorders, adjustment disorders, and anxiety disorders

		B	p	OR	95% CI for OR	
					Lower	Upper
Mood disorders	Marital status (Married/not married)	−0.849	0.014	2.08	0.218	0.841
	Education level (≤8 years / > 8 years)	0.679	0.045	1.97	1.014	3.839
	Income level (Low/Middle or upper)	−1.485	0.003	4.41	0.089	0.609
	Social Support (Poor/Sufficient)	−1.043	0.002	2.84	0.183	0.679
	Recurence (No/Yes)	−3.679	< 0.001	40	0.010	0.066
	Metastasis (No/Yes)	−1.344	0.007	3.83	0.098	0.693
	Secondary cancer (No/Yes)	−2.310	0.001	10.1	0.026	0.376
	Other medical illnesses (No/Yes)	−1.511	< 0.001	4.52	0.117	0.417
	History of psychiatric disorder (No/Yes)	−1.788	< 0.001	5.99	0.075	0.373
	Constant	7.291	< 0.001	146.7		
	Nagelkerke R^2^	0.571				
Adjustment Disorders	Marital status (Not married/Married)	−0.464	0.050	1.59	0.394	1.003
	Income level (Low/Middle-upper)	−1.256	< 0.001	3.86	0.141	0.476
	Social Support (Poor/Sufficient)	−0.452	0.030	1.57	0.424	0.957
	Duration of illnesses (0–12 / ≥13 months)	0.706	0.001	2.03	1.300	3.156
	Metastasis (No/Yes)	−0.870	0.002	2.39	0.243	0.721
	Recurence (No/Yes)	1.256	< 0.001	3.51	0.142	0.573
	Cancer history in family members (No/Yes)	−0.766	0.015	2.15	0.250	0.863
	Other medical illnesses (No/Yes)	−0.696	0.005	2.01	0.307	0.807
	History of psychiatric disorder (No/Yes)	−1.431	< 0.001	4.18	0.126	0.455
	Constant	9.631	< 0.001	152.3		
	Nagelkerke R^2^	0.276				
Anxiety Disorders	Income level (Low/Middle-upper)	0.954	0.001	2.6	1.462	4.609
	Social Support (Poor/Sufficient)	0.621	0.003	1.86	1.234	2.808
	Duration of illnesses (0–12 / ≥13 months)	0.504	0.02	1.65	1.081	2.533
	Recurence (No/Yes)	1.884	< 0.001	6.58	3.065	14.14
	Secondary cancer (No/Yes)	1.632	0.01	5.11	1.470	17.79
	Other medical illnesses (No/Yes)	0.631	0.011	1.88	1.155	3.06
	History of psychiatric disorder (No/Yes)	1.202	< 0.001	3.32	1.743	6.345
	Constant	−11.35	< 0.001	128.3		
	Nagelkerke R^2^	0.295				

*OR* Odds ratios

*CI* Confidence Intervals

Significant risk factors for adjustment disorders covered cancer recurrence, having other chronic medical illnesses, history of psychiatric disorder, the duration of illness less than 1 year, cancer history in family members, low income level, poor social support, and being single or divorced. This model explained that adjustment disorders accounted for 28% risk factors for patients, which is useful to predict the evolution of adjustment disorders (Table 3).

The risk factors identified for anxiety disorders were as follows: recurrence of cancer, presence of secondary cancer, having another chronic medical illness, history of psychiatric disorder, duration of illness less than 1 year, low income level, and poor social support. This model identified 29.5% of risk factors for patients diagnosed with anxiety disorders (Table 3).

### Risk factors according to sex for mood disorders, adjustment disorders, and anxiety disorders

For female cases, significant risk factors that increased the mood disorders were recurrence, presence of secondary cancer, other chronic medical illnesses, history of psychiatric disorder, low income level, poor social support, and being single or divorced. The model accounted for 49% of the risk factors for women diagnosed as having mood disorders. When the risk factors related to adjustment disorders in female cases were examined, recurrence, metastasis, history of psychiatric disorder, presence of other chronic medical illnesses, low income level, poor social support, and the duration of illness less than 1 year were significant risk factors that increased the likelihood of being diagnosed as having adjustment disorders. These results revealed risk factors for 25.6% of women diagnosed as having adjustment disorders. The significant risk factors that increased the likelihood of being diagnosed as having anxiety disorder were recurrence, metastasis, presence of other chronic medical illness, history of psychiatric disorder, the duration of illness less than 1 year, low income level, and poor social support. These results accounted for 30% of women diagnosed with anxiety disorders in terms of risk factors (Table 4).

**Table 4:** Risk factors according to sex for mood disorders, adjustment disorders, and anxiety disorders

		Female					Male				
					95% CI for OR					95% CI for OR	
		B	p	OR	Lower	Upper	B	p	OR	Lower	Upper
Mood disorders	Income level (Low/Middle- upper)	−1.064	0.042	2.90	0.123	0.964	−2.592	0.035	13.3	0.07	0.836
	Social Support (Poor/Sufficient)	−0.935	0.013	2.54	0.188	0.819	−1.771	0.022	5.88	0.04	0.771
	Metastasis (No/Yes)	–	–	–	–	–	−2.842	0.050	17.2	0.03	0.997
	Recurence (No/Yes)	−3.441	< 0.001	31.25	0.011	0.092	−5.495	< 0.001	250	0.01	0.065
	Secondary cancer (No/Yes)	−1.940	0.030	6.94	0.025	0.827	−3.445	0.011	31.25	0.02	0.451
	Other medical illnesses (No/Yes)	−0.984	0.009	2.67	0.178	0.785	−2.698	< 0.001	14.92	0.018	0.257
	History of psychiatric disorder (No/Yes)	−1.560	0.001	4.76	0.083	0.530	−2.135	0.017	8.47	0.021	0.679
	Constant	5.703	< 0.001	299.8			9.888	< 0.001	1970		
	Nagelkerke R^2^	0.475					0.714				
Adjustment Disorders	Education level (≤8 years/ > 8 years)	–	–	–	–	–	−1.017	0.016	2.76	0.158	0.830
	Income level (Low/Middle- upper)	0.902	0.009	2.46	1.245	4.841	2.517	< 0.001	12.39	3.146	4.78
	Social Support (Poor/Sufficient)	0.537	0.034	1.71	1.042	2.806	–	–	–	–	–
	Metastasis (No/Yes)	–	–	–	–	–	–	–	–	–	–
	Recurence (No/Yes)	1.220	0.003	3.38	1.494	7.682	2.153	0.007	8.61	1.812	4.94
	Other medical illnesses (No/Yes)	0.783	0.006	2.19	1.247	3.842	–	–	–	–	–
	Cancer history in family members (No/Yes)	–	–	–	–	–	−1.668	0.032	5.29	0.041	0.870
	History of psychiatric disorder (No/Yes)	1.212	0.003	3.36	1.518	7.440	1.459	0.017	4.303	1.302	4.219
	Constant	−2.808	0.005	16.6			−2.669	0.007	8.92		
	Nagelkerke R^2^	0.199					0.376				
Anxiety Disorders	Income level (Low/Middle- upper)	−0.767	0.027	2.15	0.235	0.918	−0.767	0.027	2.15	0.235	0.918
	Social Support (Poor/Sufficient)	−0.645	0.014	1.9	0.314	0.876	–	–	–	–	–
	Metastasis (No/Yes)	−0.893	0.021	2.43	0.192	0.876	–	–	–	–	–
	Recurence (No/Yes)	−2.052	< 0.001	7.81	0.048	0.343	−1.691	0.028	5.43	0.41	0.832
	Secondary cancer (No/Yes)	−1.502	0.048	4.06	0.049	1.229	−2.585	0.031	13.33	0.07	0.789
	Other medical illnesses (No/Yes)	−0.643	0.034	1.09	0.290	0.951	–	–	–	–	–
	History of psychiatric disorder (No/Yes)	−1.502	0.001	4.48	0.095	0.520	–	–	–	–	–
	Constant	5.591	< 0.001	167.84			3.737	0.01	41.97		
	Nagelkerke R^2^	0.280					0.316				

*OR* Odds ratios

*CI* Confidence Intervals

Significant risk factors that increased the likelihood of being diagnosed with mood disorders in male patients were recurrence, presence of secondary cancer, other chronic medical illness, history of psychiatric disorder, metastasis, being single or divorced, poor social support, and low income level. The model explained 71.5% of risk factors for men diagnosed as having mood disorders. When the risk factors for adjustment disorders in male patients were examined, low income level, recurrence, cancer history in family members, and history of psychiatric disorder were identified as significant risk factors for adjustment disorders. The model defined 38.4% of risk factors in men diagnosed as having adjustment disorders. For men with the diagnosis of anxiety disorders, low income level, and recurrence or the presence of secondary cancer were identified as significant risk factors. The model explained the risk factors of 30% of men diagnosed as having anxiety disorders (Table 4).

## Discussion

Offering mental health services to patients with cancer is becoming an integral part of oncologic treatments because psychological problems have an adverse effect on cancer management. The main aim of this study was to provide a comprehensive description of baseline information on patients with cancer seeking help for mental health and to define the overall and sex-specific risk factors for the 3 main diagnostic groups (mood disorders, adjustment disorders, anxiety disorders) at the outpatient psycho-oncology clinic over a 2-year period.

The rate of women who presented to the outpatient department of psycho-oncology was determined to be significantly higher than for men (Table 1). The number of women with breast cancer who presented to our department was high. It is known that women with breast cancer may experience intense psychological difficulties caused by the negative mental effects of the disease itself and the treatment processes including surgery, chemotherapy, hormone therapy or radiotherapy [21, 22]. Another explanation may be that the women are more inclined to express their problems and are keener to seek psychiatric help, whereas men suppress their emotions more and therefore abstain from seeking psychological help. It has been reported that women seek more help among patients with cancer who are followed up in outpatient departments [23]. In traditional Turkish society, men feel that they are meant to present a strong/resistant image, which may have restricted male patients from expressing emotions and seeking help. The reason why the mean age of the women was significantly lower than for male patients may be due to the fact that women with breast cancer comprise a large portion of the population presenting to our department (Table 1). It has been reported that 61.5% of women with breast cancer were aged between 19 and 54 years in Turkey [24].

Of the help-seeking patients with cancer who presented to our department, 97.5% received psychiatric diagnoses, which is very high compared with the 59.6% reported in the literature [10]. The high rate of psychiatric diagnoses may be due to the fact that the patients referred to our department already had noticeable psychological problems, which means that they should have sought help earlier. The main reason for patients with cancer withholding their depression and anxiety during oncology visits is that they want to spend their limited time talking about the important issues associated with their illnesses [25]. Therefore, oncologists should inquire into the emotional status of patients, even if they do not mention any psychological challenges.

Another reason for delaying seeking help may be that they suppose they can cope with their psychological problems on their own. Clover et al. (2015) reported that 46% of patients with psychological distress had no help for treatment because they preferred to manage the process themselves [23]. Prejudice against receiving psychiatry or psychology treatment can be a major source of resistance in our culture. From the point of view of patients, prejudices such as “only insane people receive treatment from psychiatrists or psychologists” may prevent them from receiving help for psychological problems. At the same time, from the standpoint of physicians, the concern that patients may react negatively to referral to psycho-oncology department may prevent physicians from referring them. As reported in the literature, cultural barriers to which patients are exposed, which impede oncologists’ referral of patients to seek help for mental health, should not outweigh the seriousness of psychological problems [26]. The lack of knowledge of how to approach psychological problems, whether there is a real problem experienced by the patient, and the workload and time limitations of oncology staff are among the most important discouraging factors [27, 28].

When examining the charts of patients, we noticed that oncologists directed the majority of the patients to the outpatient department of psycho-oncology (Table 2). Mackenzie et al. (2015) support our findings, that the majority of patients with cancer choose to report their concerns and depression to their medical practitioners [25]. Informing physicians about psycho-oncology practices and maintaining active cooperation in oncology is important in terms of providing mental health services more effectively.

The symptoms that lead patients to our outpatient department include sleep problems, irritability, tendency to cry easily, depression, and pain (not compatible with the medical condition) (Table 2). Women reported sleeping problems, tendency to cry easily, irritability, pre-occupation with illness, and despondency as the most frequent symptoms, and men reported sleep problems, irritability, pain, despondency, and a tendency to cry easily (Table 2). Sleep problems were noted to be the most prominent symptoms of depression, adjustment disorders, and anxiety; the rate of patients with cancer diagnosed as having insomnia only is reported as high as 30–60% [28, 29].

The distribution of the prevalence of psychiatric diagnoses was as follows: adjustment disorder (65%), and mood disorders (18%) (e.g. major depression, bipolar disorder, complicated mourning), anxiety disorders (9.7%), organic brain syndrome (1.4%), personality disorder (1.4%), delusional disorder (1.2%), and insomnia (0.9%), as well as comorbidities (depression associated with either generalized anxiety disorder or obsessive compulsive disorder), which is compatible with the results reported in the literature (Table 2). Derogatis et al. stated that 68% of patients with cancer had adjustment disorder, 13% major depression, 8% organic brain syndrome (OBS), 7% personality disorder, and 4% pre-existing anxiety disorder [7]. Adjustment disorders in patients with cancer have been reported as 55.8% in Turkey [30]. Patients with cancer have 10–25% major depression and clinically significant depressive symptoms at similar rates [17, 30]. Tokgöz et al. found that depression prevalence was 22% in patients with cancer and sleep problems were frequent [31]. The fact that we reported a small number of patients with OBS might be due to (i) the study covered only outpatient patients who actively sought help (ii) patients with OBS usually present to the emergency department of psychiatry.

According to the results of the regression analysis, the common risk factors for adjustment disorders, mood disorders, and anxiety disorders were low income level, poor social support, cancer recurrence, other chronic medical illness, and history of psychiatric disorder (Table 3). Our findings are consistent with the risk factors reported for psychiatric disorders in patients with cancer [16, 32, 33]. In this study, the most significant risk factor for all diagnostic groups was the recurrence of cancer; metastasis was not found as a significant risk factor for any of the diagnostic groups. At this point, it is necessary to discuss why disease recurrence has a much greater adverse effect than metastasis on mental health. Burgess et al. found that 222 women with early breast cancer had depression and anxiety disorders at a rate of 33% during the course of diagnosis, at a rate of 15% at 1 year, and at a rate of 45% during recurrence of diseases [34]. The reason why patients develop depression or anxiety upon learning about the recurrence of cancer may arise from experience of former therapeutic processes (chemotherapy, radiotherapy or surgery), which is not surprising. We think that metastasis was not such a significant factor for all three diagnostic groups because the patients might be unaware of metastasis or what metastasis means or were not informed about metastasis. This is because medical professionals do not have to legally inform patients about the diagnosis and disease progression, and sometimes the family does not want the patient to be informed about the cancer in order to protect the patient [35, 36].

Our findings are consistent with the literature in that the risk for adjustment disorder and anxiety disorders increases when the patients receive cancer diagnosis, particularly in the first year [37]. There were no differences in risk factors for any psychiatric diagnoses with respect to age and sex in our study. In contrast to our findings, some studies have reported a significant sex or age difference in the cancer population [31, 38, 39], but there are findings compatible with ours in the medical literature [40, 41]. Age and sex might not seem to have been significant risk factors in our study because it included patients with a wide range of cancer types and stages.

When we examined the risk factors in terms of sex in the psychiatric diagnosis groups, it was determined that disease recurrence, history of psychiatric disorder, and low income level were common risk factors for both men and women. The presence of other chronic medical illnesses and poor social support, which were found as significant risk factors for the three diagnostic groups, were identified as leading significant risk factors for women. Metastasis was identified as a significant risk factor for anxiety disorders in women and for mood diagnoses in men. As seen, the risk factors determined for the overall patient population and the risk factors determined on the basis of sex are similar in many respects but differ in some points (Table 4). Our study describes a large number of patients seeking help for mental health with various types of cancer in terms of sociodemographic characteristics, disease characteristics, and psychiatric characteristics. More importantly, it draws a profile of risk factors for severe mental problems that we encountered in both overall risk factors and sex-related risk factors in patients with cancer seeking help for mental health. In addition, the generalizability of the data obtained in the regression analysis is very high – approximately 50% in women, 70% in men – for mood disorders.

### Clinical implications

This paper gives a clear picture of patients with cancer who seek help for mental health by presenting information on characteristics (sociodemographic, cancer-related, and psychiatric), the prevalence of mental disorders, as well as risk factors for mood disorders, adjustment disorders, and anxiety disorders. When there is a delay – for whatever reason – in seeking help for mental health, undesirable consequences such as increased morbidity, noncompliance with treatment, increasing mortality may appear [41]. Based on this evidence, the fact that 97.5% of our patients received diagnoses of psychiatric disorders shows how valuable early referral could be. Our study will also contribute to raising oncologists’ awareness toward the various risk factors, thus more patients at risk for mental disorders can be identified by oncologists and more patients may gain access to mental health care earlier.

Our study results involve all types and stages of cancers and both sexes; therefore, they could be used in developing and planning effective psycho-oncology services and guide research in the field of psycho-oncology. Additionally, the study raises questions that draw attention to patients’ mental distress in oncology practice:

1. What are the characteristics of patients who do not seek/ demand help but are at a high risk for mental health problems?
2. What are the potential drawbacks of patients with cancer seeking help in terms of the patients themselves, environmental and contextual, and how can we handle such challenges?
3. How can psycho-oncology services be further improved to be extended to patients with cancer in general?

### Study strengths and limitations

This study has some strengths and limitations. One of the strengths was that our sample consisted of patients with cancer with various stages and types of disease who presented to the outpatient psycho-oncology clinic, thus not focusing on patients with a single cancer type or stage. Our study reflects the data on outpatient patients with cancer who were referred to seek help for mental health and actively complied with the recommendations of their doctors. One limitation of the study is that it is an exploratory study with a retrospective chart review design. Another is the lack of assessment of important psychological aspects, such as coping styles and quality of life. A further limitation is that, although DSM-IV-TR diagnostic criteria were used for diagnostic evaluation, the possibility of individual differences of practitioners could not be completely excluded. Finally, the study does not reflect inpatient data because it is limited to patients who were referred to the psycho-oncology outpatient clinic.

## Conclusion

This study identifies the distribution of psychiatric disorders, the risk factors for specific psychiatric disorders, and draws attention to the fact that there are serious delays in patients seeking psychiatric help and in the referrals of oncologists for psychological assessment. Identifying risk factors and raising oncologists’ awareness toward risk factors could help more patients gain access to mental health care much earlier. In addition, media institutions can be supported to overcome social prejudices about the need for psychological help and to raise public awareness of the psychological problems that arise in cancer. These may increase the number of patients who become aware of psychological problems and seeking help for mental help.

## Data Availability

The patient cards used in the study can not be shared with other researchers because they contain personal information (the identification number, the birth date etc.). The data sets used and analyzed during the current study are available from the corresponding author on reasonable request and with consent from the Institutional Review Board (IRB).

## List of abbreviations

C.I.: Confidence Intervals
CLP: Consultation Liaison Psychiatry
DSM-IV-TR: Statistical Manual of Mental Disorders, 4th Edition
OR: Odds ratios
